# Accurate comparison of wearables requires contextual equivalence

**DOI:** 10.14814/phy2.70710

**Published:** 2025-12-13

**Authors:** Gregory J. Grosicki, David M. Presby

**Affiliations:** ^1^ Department of Performance Science WHOOP, Inc. Boston Massachusetts USA; ^2^ Department of Data Science WHOOP, Inc. Boston Massachusetts USA

We read, with great interest, the recent article by Dial et al. (2025) titled “Validation of nocturnal resting heart rate and heart rate variability in consumer wearables.” The authors present valuable data from 13 healthy adults across 536 nights of sleep to explore agreement between wearable‐ and chest‐strap‐derived measures of resting heart rate (RHR) and heart rate variability (HRV). As the use of wearable technology continues to rise, with many devices now entering health care settings, the need for transparent and methodologically sound validation studies is increasingly important (Ginsburg et al., 2024). RHR and HRV are widely used non‐invasive indices of cardiac autonomic function and are well‐established predictors of both short‐term training adaptation (Plews et al., 2013) and long‐term health risks including cardiovascular‐specific and all‐cause mortality (Fang et al., 2020; Zhang et al., 2016). This work therefore represents a valuable step towards understanding how several of the most widely used consumer wearable devices perform in capturing these key parameters during sleep.

Fundamental to accurate comparison of wearables is contextual equivalence, which considers alignment between device and reference on (i) metric definition (e.g., HRV as RMSSD vs. SDNN), (ii) temporal sampling window (e.g., first 4 h vs. whole night or stage‐specific), and (iii) the averaging or weighting procedure applied to those samples. The authors acknowledge that each wearable computes RHR and HRV using different parameters, with varying frequencies, requirements, and processes (Dial et al., 2025). In light of these differences, Dial et al. adjusted their analysis for the Polar Grit X Pro (Polar, Kempele, Finland) by matching reference data to that reported by the device (i.e., during the first 4 h of sleep) and excluded the Garmin Fenix 6 (Garmin, Kansas City, MO, USA) from RHR analysis owing to its reporting of values from the lowest 30‐min average in a 24‐h period. While the authors acknowledge that WHOOP 4.0 (WHOOP Inc., Boston, MA, USA) calculates HRV using a dynamic average during sleep weighted towards the last phase of slow wave sleep, they compared WHOOP‐derived RHR and HRV to an all‐night average from the reference chest strap, thereby potentially compromising the temporal aspect of contextual equivalence.

Recognizing the distinction between RHR and HRV metrics derived from individual sleep stages and those from whole‐night aggregates is important, given established sleep‐stage‐specific differences in RHR and HRV. For example, one study found RHR to be 3.5% lower and normalized high‐frequency HRV (HFpn) 123.7% higher during slow wave sleep (SWS) compared with rapid eye movement (REM) sleep (Trinder et al., 2001), though exact magnitudes may vary by cohort and study. While the author's use of within‐individual *z*‐scores to account for interindividual variability is commendable, this approach assumes that the relative differences between stages remain constant. However, perturbations such as stress can alter cardiac autonomic activity in a stage‐specific manner and shift the time spent in each stage (Beck et al., 2022; Hall et al., 2004), thereby introducing non‐uniform changes that z‐scoring cannot account for. Consequently, both absolute and normalized measures of RHR and HRV can differ depending on the stage of sleep from which they are derived, independent of device differences. Therefore, the resulting temporal mismatch, comparing a stage‐weighted WHOOP metric to an all‐night reference average, may bias device‐to‐device comparisons and compromise conclusions about validity across devices.

We recognize that proprietary algorithms pose a persistent challenge for validation efforts. Looking ahead, we advocate for deeper collaboration between wearable companies, clinicians, and the academic community to ensure contextual equivalence. WHOOP has previously demonstrated its support for such efforts by providing third‐party researchers with full‐night, 30‐s epoch data, which yielded excellent agreement with a reference II‐lead ECG for both RHR and HRV (ICCs = 0.99) in a mixed‐sex sample of 53 participants (Miller et al., 2022). This example illustrates the high degree of validity possible when raw data access is provided, enabling temporal alignment. Moving forward, scaling these efforts to evaluate device performance across more diverse cohorts spanning age, sex, race, and health status will be critical to improving the relevance, generalizability, and equity of wearable‐derived health metrics (Walter et al., 2024).

In conclusion, we applaud the authors for contributing to the important and evolving field of wearable validation. We respectfully recommend that future studies strive for strict contextual alignment across devices and prioritize access to temporally matched data. We also encourage expansion of validation efforts to include more demographically and clinically diverse populations. These steps, supported by transparent industry‐academic partnerships and rigorous validation efforts, will help to realize the full potential of wearable technologies to support health, performance, and clinical care.

## CONFLICT OF INTEREST STATEMENT

Gregory Grosicki and David Presby are affiliated with the commercial company WHOOP, Inc. which provided support in the form of salaries. The views expressed in this letter are solely those of the authors and do not necessarily represent those of WHOOP, Inc. WHOOP, Inc. had no role in the conception, drafting, or review of this letter and did not influence its content.
